# Bioresorbable Stent in Anterior Cruciate Ligament Reconstruction

**DOI:** 10.3390/polym11121961

**Published:** 2019-11-29

**Authors:** Krzysztof Ficek, Jolanta Rajca, Mateusz Stolarz, Ewa Stodolak-Zych, Jarosław Wieczorek, Małgorzata Muzalewska, Marek Wyleżoł, Zygmunt Wróbel, Marcin Binkowski, Stanisław Błażewicz

**Affiliations:** 1 Department of Science, Innovation and Development, Galen-Orthopaedics, 43-150 Bierun, Poland; krzysztof.ficek@galen.pl (K.F.); matstolarz@gmail.com (M.S.); 2 Department of Physiotherapy, Academy of Physical Education, 40-065 Katowice, Poland; 3 Department of Orthopedics and Traumatology, City Hospital in Zabrze, 41-803 Zabrze, Poland; 4 Department of Biomaterials and Composites, Faculty of Materials Science and Ceramics, AGH University of Science and Technology, 30-059 Krakow, Poland; stodolak@agh.edu.pl (E.S.-Z.); blazew@agh.edu.pl (S.B.); 5 University Center of Veterinary Medicine UJ-UR, University of Agriculture in Krakow, 30-059 Krakow, Poland; jaroslaw.wieczorek@urk.edu.pl; 6 Institute of Fundamentals of Machinery Design, Faculty of Mechanical Engineering, Silesian University of Technology, 44-100 Gliwice, Poland; muzalewska.malgosia@gmail.com (M.M.); marek.wylezol@polsl.pl (M.W.); 7 Institute of Biomedical Engineering, Faculty of Science and Technology, University of Silesia, 41-205 Sosnowiec, Poland; zygmunt.wrobel@us.edu.pl; 8 X-ray Microtomography Lab, Department of Computer Biomedical Systems, Institute of Computer Science, Faculty of Computer and Materials Science, University of Silesia, 41-200 Sosnowiec, Poland; binkowski.marcin@gmail.com

**Keywords:** anterior cruciate ligament reconstruction, bone tunnel enlargement, X-ray microtomography, polylactide

## Abstract

The exact causes of failure of anterior cruciate ligament (ACL) reconstruction are still unknown. A key to successful ACL reconstruction is the prevention of bone tunnel enlargement (BTE). In this study, a new strategy to improve the outcome of ACL reconstruction was analyzed using a bioresorbable polylactide (PLA) stent as a catalyst for the healing process. The study included 24 sheep with 12 months of age. The animals were randomized to the PLA group (n = 16) and control group (n = 8), subjected to the ACL reconstruction with and without the implantation of the PLA tube, respectively. The sheep were sacrificed 6 or 12 weeks post-procedure, and their knee joints were evaluated by X-ray microcomputed tomography with a 50 μm resolution. While the analysis of tibial and femoral tunnel diameters and volumes demonstrated the presence of BTE in both groups, the enlargement was less evident in the PLA group. Also, the microstructural parameters of the bone adjacent to the tunnels tended to be better in the PLA group. This suggested that the implantation of a bioresorbable PLA tube might facilitate osteointegration of the tendon graft after the ACL reconstruction. The beneficial effects of the stent were likely associated with osteogenic and osteoconductive properties of polylactide.

## 1. Introduction

The causes of failure of anterior cruciate ligament (ACL) reconstruction are still a matter of debate. While many various ACL reconstruction techniques have been proposed thus far, none of them seems to be optimal and complication-free [1,2,3]. The failures of ACL reconstruction might be associated with an inappropriate orientation of bone tunnels, use of improper fixation methods and materials, and inadequate rehabilitation, as well as with mechanical behavior of the bone and biological processes that occur during remodeling, maturation, and incorporation of the graft [4,5]. The healing potential of a newly implanted graft is relatively low [6,7,8] and is primarily determined by conditions within proximity of the bone tunnel and soft tissue of the graft, including the intra-articular environment. Osteointegration of the tendon grafts used for ACL reconstruction is still far from satisfactory, although several strategies have been postulated to improve the process [9,10,11,12,13,14,15,16,17,18,19].

Another critical determinant of successful ACL reconstruction is the prevention of bone tunnel enlargement (BTE), a phenomenon of mechanical and biological etiology. The mechanical causes of BTE might be related to the tunnel drilling technique, graft fixation technique, vibrations at the tunnel entry, and movements of the graft referred to as “bungee effect” and “windshield wiper effect” [20,21,22,23,24,25]. The biological mechanisms involved in the BTE include accumulation of intra-articular fluid, which penetrates to the space between the graft and the wall of the bone tunnel. The sites in which the graft is not adjacent closely to the bone tunnel wall, the so-called “dead space”, are particularly prone to fluid accumulation. The intra-articular fluid that accumulates after the ACL rupture contains proinflammatory cytokines, which are responsible for local osteolysis [26,27,28,29]. Another biological mechanism implicated in BTE pathogenesis might be the so-called “synovial bathing effect”; due to its excessive accumulation after ACL reconstruction, synovial fluid might be pressed into the bone tunnel and cause enlargement thereof [23,30,31].

Polylactide (PLA) is a polymer used in surgical practice for bone anastomosis implants (screws, plates, rods). PLA owes its popularity not only to its good biocompatibility (the best among polymers) but also to the ease of implant formation (injection, extrusion, printing). Polylactide is a biodegradable polymer, the durability of which can be determined in vivo by in vitro degradation tests. Degradation of polylactide was a subject of many published studies [32,33,34]. According to some authors, the degradation of PLA in strongly hydrated environments occurs through the penetration of water molecules and hydrolysis of ester bonds, which results in an increase in the concentration of terminal carboxylic groups [35]. Other researchers have suggested that the PLA polymer chain is autocatalyzed due to acidification of the environment (dissociation effect of carboxyl groups) [36]. The result of material degradation is a decrease in molecular weight and a larger dispersion of average molecular weight. The kinetics of the process depends on the structure of the polymer chain, especially the presence of L or D enantiomer and their ratio [34]. Many methods can be used to control the degradation process in order to reduce the negative effect of PLA autocatalysis. One of them is the addition of modifiers capable of connecting hydronium ions to the polymeric matrix [37], as it is the case with PLA-based nanocomposites modified with ceramic particles. Unfortunately, in the case of new materials, many other parameters need to be tested, and validated in vitro and in vivo tests are required. Therefore, it seems easier to use a minimum amount of pure PLA, e.g., in the form of membranes or highly porous materials, especially when their task is not limited to the transfer of stress. An example of such a solution is presented below. The perforated microscopic material also has pores at the microstructural level, thus reducing the total amount of material used to produce the implant. The presented solutions significantly affect the durability of the material in vitro, which allows approximating the time of degradation in vivo.

**Figure A2:**
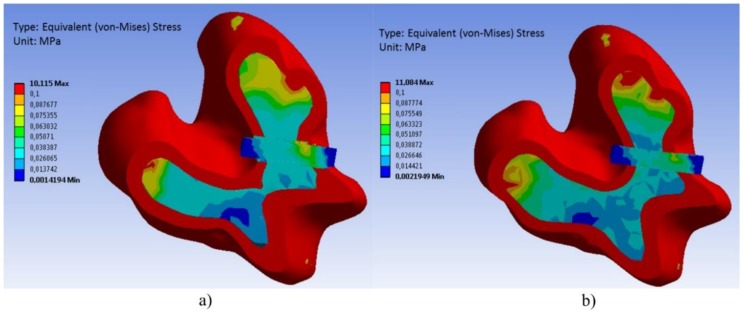
Stress distribution within the femur: (**a**) with a stent, (**b**) without a stent.

**Figure A3:**
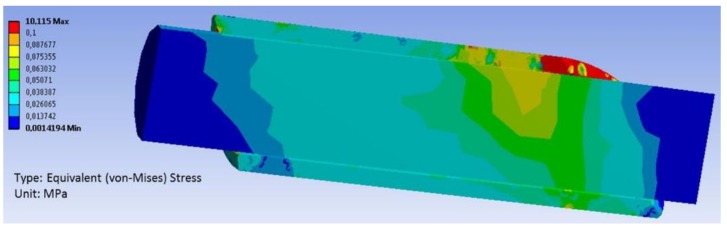
Stress distribution within the graft and stent.

**Figure A4:**
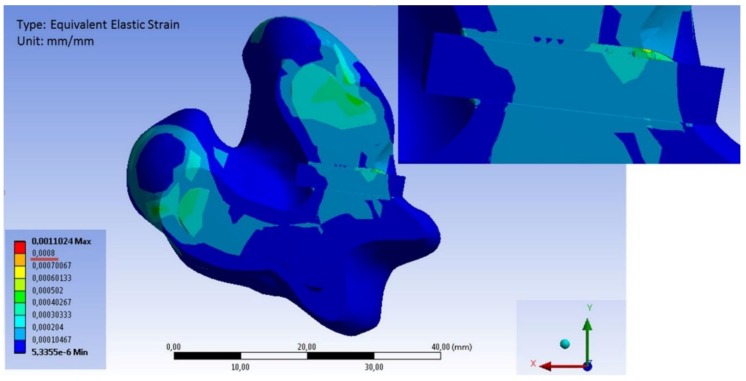
Strain distribution within the femur with the stent.

**Figure A5:**
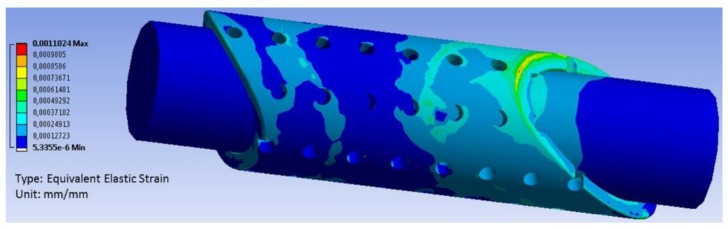
Strain distribution within the graft and stent.

In our present study, we analyzed another strategy to improve the outcome of ACL reconstruction, using a bioresorbable polylactide (PLA) stent as a catalyst for the healing process. The stent was produced from poly(L/DL-lactide) 80/20 (80:20 PL/DLA is name of molar ratio the combination of L-lactate (80%) and DL-lactate (20%)) [38,39,40,41,42]. Bioresorbable PLA polymers, in the form of powder, beads, or paste, have already been tested in sheep [39,41], rabbits [42,43], and humans [41,43]. However, applied in a non-solid form, PLA did not provide sufficient mechanical support, which eventually contributed to BTE. Thus, in this study, we verified whether PLA in another form, a tube-shaped perforated stent with a porosity of 45%, improved graft-bone integration after ACL reconstruction. We hypothesized that implantation of the stent could accelerate the bone healing process and prevent the accumulation of non-uniform forces inside the bone tunnel, reducing the risk of BTE. This hypothesis was first verified in a virtual environment, based on finite element analysis of the stress-strain response from the bone, graft, and PLA stent (see: Appendix A, Figure A2, Figure A3, Figure A4 and Figure A5). The results constituted the basis for the proper animal experiment, the results of which are described below. The microarchitecture of bone tunnels and adjacent bone was analyzed based on high-resolution, high-quality images obtained during X-ray microtomography (micro-CT). The study involved sheep, as this animal was previously shown to be a good model for orthopedic research [44,45].

## 2. Materials and Methods

### 2.1. Animals and Specimen Preparation

The study included 24 male sheep with 12 months of age and body weights ranging between 35 and 40 kg. The protocol of the study was approved by the Animal Ethics Committee at the Institute of Pharmacology, Polish Academy of Sciences in Krakow (decisions no. 820/2011 and 836/2011 of 27 January 2011 and 19 May 2011, respectively). The animals were randomized into two groups: the PLA group (n = 16) and the control group (n = 8). The sheep were kept under standard husbandry conditions (stalls with straw bedding, temperature 10–25 °C, natural light/dark cycle according to the season, with 8 to 16 h lights on), two animals per stall, matched according to the experimental group. The animals were fed with hay (ad libitum) and pasture (0.3 kg/animal/day) and had unlimited access to water. The food was restricted one day before the ACL reconstruction procedure.

**Figure 1:**
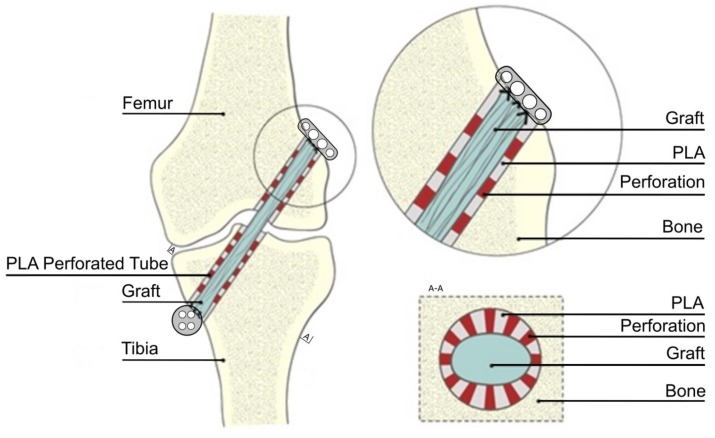
Scheme of the ACL reconstruction, type of graft fixation, and the implantation of polylactide (PLA) perforated tube.

The reconstruction procedure was carried out under general anesthesia by inhalation nitrous oxide 25–40% (Linde Gas, Krakow, Poland), isoflurane 0.6–3% (Aerrane 250 mL, Baxter, Warsaw, Poland), and oxygen 30–40% (Medical Oxygen, Linde Gas, Warsaw, Poland). First, the animal’s ACL was cut and removed from the joint. Then, tibial and femoral bone tunnels, 4.5 mm in diameter, were drilled using the ACL insertions as the landmarks (Figure 1). The tunnels were drilled in the medial aspect of the proximal tibial metaphysis and distal femoral metaphysis lateral to the condyles. The autograft was harvested from the Achilles tendon. In the control group, the autograft, 4.5 mm in diameter, was pulled through both bone tunnels, and then, its ends were fixed extracortically to the femur and tibia with an EndoButton and button (Figure 1). In the PLA group, PLA tubes (3.5 mm in diameter) were first placed in the tunnels, and then, the autograft, also 3.5 mm in diameter, was inserted into the tubes, so it adhered tightly to their inner walls. Finally, the graft’s ends were fixed analogically, as in the control group.

After the procedure, the sheep were placed in stalls and allowed to move freely. The animals were controlled on a daily basis for 6–12 weeks. Twelve sheep, four from the control group and eight from the PLA group, were sacrificed at 6 weeks post-reconstruction, followed by another 12 sheep, four from the control group and eight from the PLA group, at 12 weeks after the procedure. The animals were euthanized with pentobarbital sodium (Morbital, Biovet, Pulawy, Poland) overdose (50–80 mg/kg), and the previously operated knee joints were dissected to enable accurate examination of the bone tunnels.

### 2.2. Polylactide Tubes

**Figure 2:**
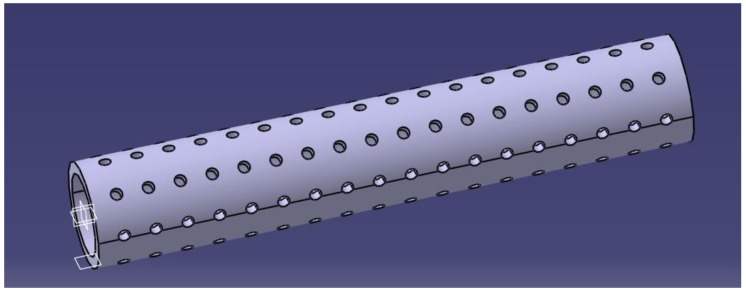
PLA perforated tube.

The PLA stents were produced from bioresorbable poly(L/DL-lactide) 80/20 (PURAC Biochem, Gorinchem, The Netherlands). The tubes were prepared using a thermal method. Briefly, the polymer pellets were heated to 156 °C, which is close to the melting point for PLA, and shaped by compression in a cylindrical metallic form to obtain a polymer film with approximately 500 μm thickness. Each PLA tube, 3.5 mm in diameter and 25 mm in length, had twenty circular-shaped perforations (0.5 mm in diameter) per square cm (Figure 2). The perforations were made with a laser device. Upon manufacturing, the PLA tubes underwent low-temperature plasma sterilization (H_2_O_2_, 40 °C).

**Figure 3:**
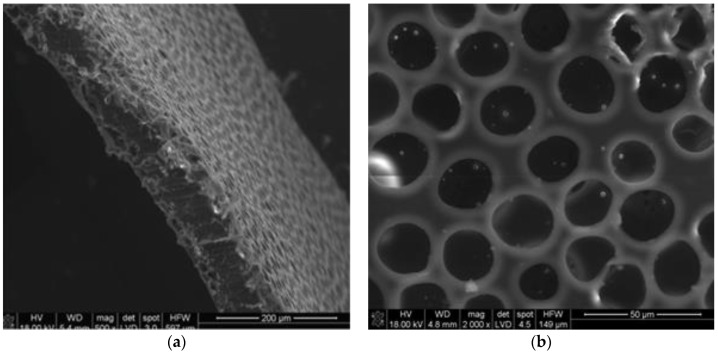
The microstructure of the polymer membranes used to form implants in the form of tubes. (**a**) membrane cross-section, (**b**) membrane surface.

Detailed methodology of polymer membrane production and the results of functional testing can be found elsewhere [46,47]. Briefly, polymer membranes were produced from a synthetic PL/DLA polymer, using a phase inversion method. The flat membrane used later for the production of the stent was manufactured by casting. A combination of tetrahydrofuran and acetone in a 10:1 ratio was used as a solvent. The polymer was homogenized for 3 h to obtain a homogeneous solution (3% *w/v*). Then, a porogenic agent, dimethylsulfoxide (DMSO), and pure water (UHQ) were added in a 1:1 ratio. The suspension was homogenized for a few minutes, poured onto Petri plates, dried, and conditioned. Then, the membranes obtained, as described above, were bathed in ethyl alcohol to remove the remains of DMSO. The final porosity of the membrane was 45%. The pore distribution in the membrane was binomodal, proving the existence of two-pore populations: the first one, more numerous with the size of about 26 µm (85%), and the second one with the average size of about 7 µm (10%). These values were determined on the basis of microscopic image analysis obtained with a scanning electron microscope (Nova NanoSEM, FEI, Hillsboro, OR, USA)) (Figure 3). The physicochemical properties of the membranes were determined based on their roughness and wettability tests. The influence of the in vitro environment on the durability of the membrane’s material was monitored based on pH alterations in water and phosphate-buffered saline extracts. In line with the standard requirements for degradability testing, the membranes were kept in an incubator for 4 weeks. The molecular weight of the membrane at the end of the experiment was determined with an Ubbelohde viscometer, with norm DIN 51562, SI Analytics, Germany (measuring liquid tethrahydrofurane, *K* = 4.85 × 10^−4^ dL/g and *a* = 0.68). The degradability of the membrane was also monitored *in vitro* (three months/H_2_O/37 °C/5% CO_2_ incubation); over the three-month monitoring period, the mean molecular weight of the membrane determined with an Ubbelohde viscometer decreased from 200 to 145 kDa. The pH of the immersion medium decreased slightly during the monitoring period, down to 6.2.

### 2.3. Micro-CT Scanning

**Figure 4:**
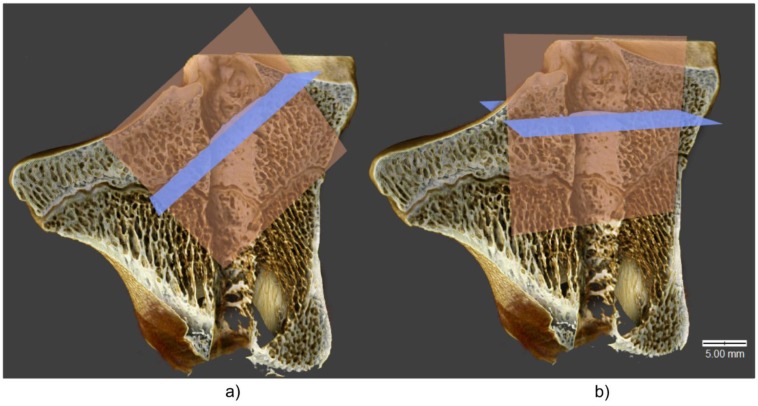
Three-dimensional visualization of the tibia. Axial cross-section—blue, sagittal cross-section—orange; (**a**) before reslicing, (**b**) after reslicing.

The knee joints from 24 sheep were scanned at the X-ray Microtomography Lab (XML), Institute of Computer Science, University of Silesia (Sosnowiec, Poland), using an XMT scanner (v|tome|x s, GE Sensing and Inspection Technologies, Phoenix|x-ray, Wunstorf, Germany). The X-ray images were acquired using a 140 kV voltage, 350 mA current, and 50 μm resolution. To accurately analyze the regeneration of the tibial and femoral tunnels on micro-CT, the images underwent reslicing so that the long central axis of each image corresponded to the exact center of the bone tunnel. An example of reslicing and image transformation, with axial cross-sections perpendicular to the bone tunnel’s axis of symmetry, is shown in Figure 4. All advanced image analyses were carried out with ImageJ, an open-source image enumeration software package (US National Institute of Health, Bethesda, MD, USA) [48].

**Figure 5:**
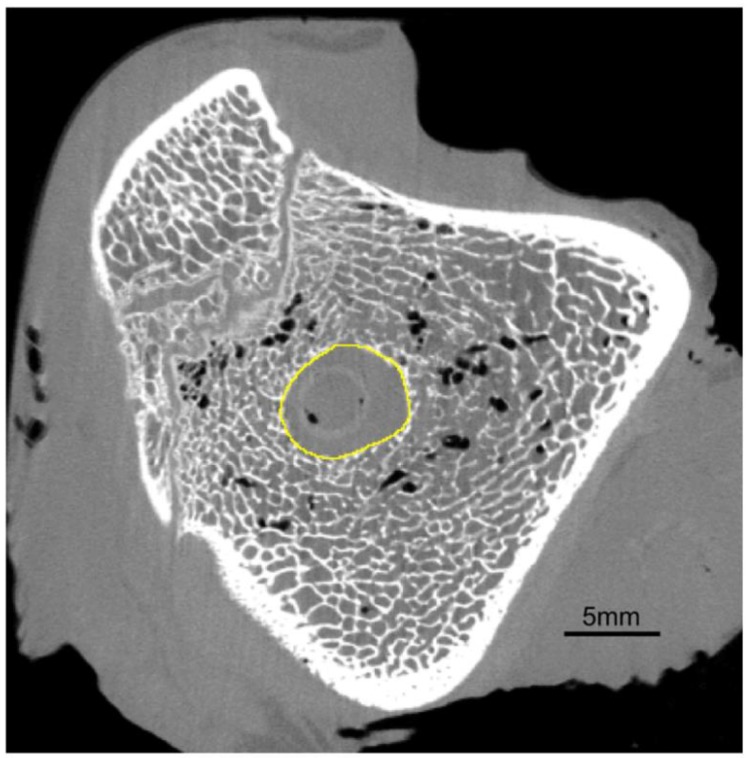
Axial cross-section of the tibia; yellow—a selection of ROI (regions of interest).

The analysis began with the selection of the tunnel’s edge on each axial cross-section of the tibia and femur (Figure 5). The boundary between the intra-tunnel space and the bone was chosen manually, to obtain an optimal area in the variable portion of the bone tunnel. To avoid a time-consuming selection of edges on each slice, the operator marked the edges on several slices, and then, the regions of interest (ROIs) were interpolated onto the remaining slices.

### 2.4. Analysis of the Tunnel Diameter After the ACL Reconstruction

**Figure 6:**
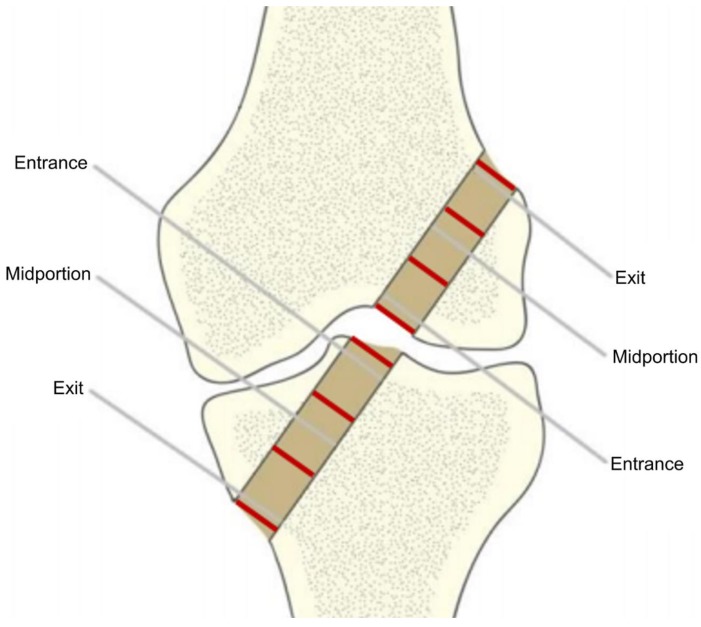
Division of bone tunnels for the analysis.

The diameter of the bone tunnel in the axial cross-section (Figure 5) was estimated by computing the diameter of the circle fitted automatically into the selected ROI of each slice, using ImageJ 1.49b software (Wayne Rasband National Institutes of Health, Bethesda, MD, USA) [48]. Then, mean diameters were calculated for three segments of the tunnel: entry, midportion, and exit (Figure 6) using pre-specified ROIs. The three segments of the tunnel were defined by dividing its entire length into three equal parts.

### 2.5. Measurement of Tunnel Volume and Determination of Histomorphometric Parameters

**Figure 7:**
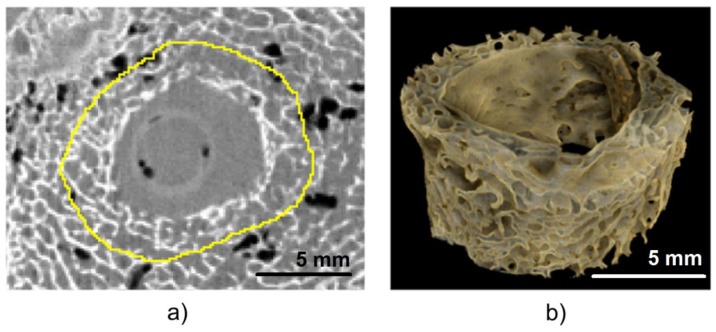
Region of interest for the analysis of histomorphometric parameters. (**a**) axial cross-section with the boundary of the ROI magnified three times—yellow line, (**b**) visualization of the segmented bone tissue.

To examine the entry of bone tunnel in more detail, its volume and histomorphometric parameters of the adjacent bone were determined. The measurements were taken inside the tunnel, 5 mm from its entry. Tunnel volume was calculated using the previously defined ROIs. Histomorphometric parameters of the adjacent bone, such as bone volume fraction (BV/TV), trabecular thickness (Tb.Th), and connectivity density (Conn.D), were determined based on micro-CT images. To obtain the measurements of trabecular structures, the area inside each ROI (Figure 5) was increased three times (Figure 7a,b). The maximum entropy method, an automated global thresholding method, was proposed for the segmentation of bone samples in our study; in this method, the threshold was chosen based on maximizing the inter-class entropy. Then, different automated algorithms were tested to select the most appropriate method for the segmentation of trabecular bone in sheep. Bone volume fraction (BV/TV), calculated as bone volume divided by total volume, corresponds to the percentage of mineralized bone located within the volume of interest (VOI). Trabecular thickness (Tb.Th) provides information about the thickness of trabecular structures. Connectivity is defined as the maximum number of trabecular connections that can be severed before the structure is split into two separate parts [49]. All three parameters were calculated using the BoneJ plugin [50] within the ImageJ software.

### 2.6. Three-Dimensional Visualization

**Figure 8:**
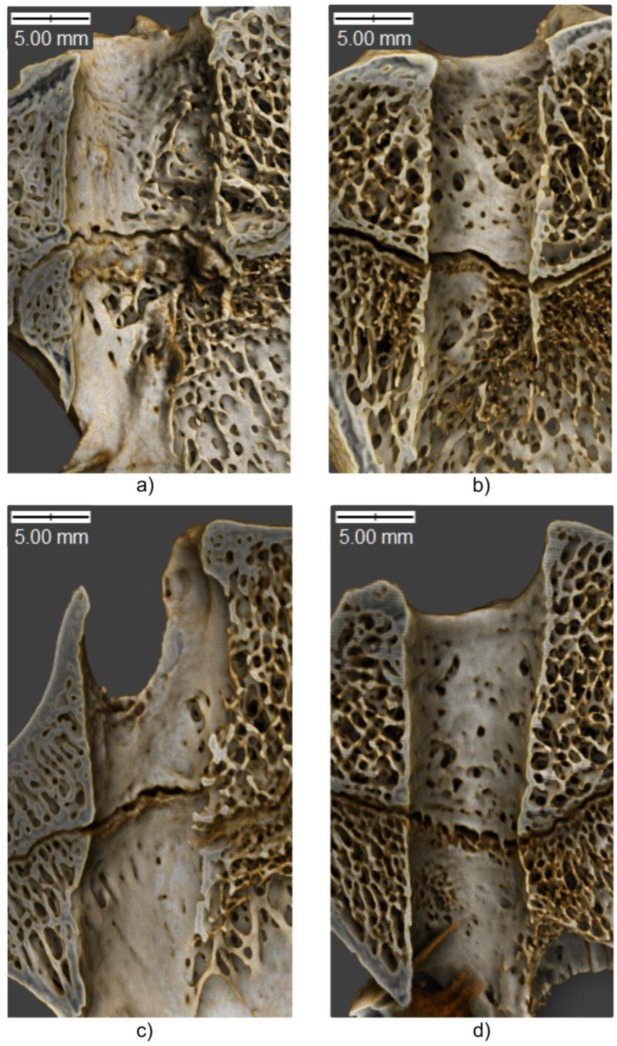
Three-dimensional visualization of the tibial tunnel in sagittal cross-section. (**a**) control at 6 weeks, (**b**) PLA at 6 weeks, (**c**) control at 12 weeks, (**d**) PLA at 12 weeks.

The changes in histomorphometric parameters of adjacent bone were visualized using Drishti open-source software [51]. On three-dimensional images (Figure 8), the bone tunnels were presented in sagittal cross-section, with the extracortical button placed at the bottom left side of the image.

### 2.7. Statistical Analysis

Normal distribution of the study variables was verified with the Shapiro–Wilk test, and the equality of their variances was checked with Levene’s test. The significance of intergroup differences was verified with non-parametric Mann–Whitney U-test. All calculations were carried out with Statistica 10 (StatSoft, Tulsa, OK, USA), with the threshold of statistical significance set at *p* < 0.05.

## 3. Results

### 3.1. Bone Tunnel Measurements

**Figure 9:**
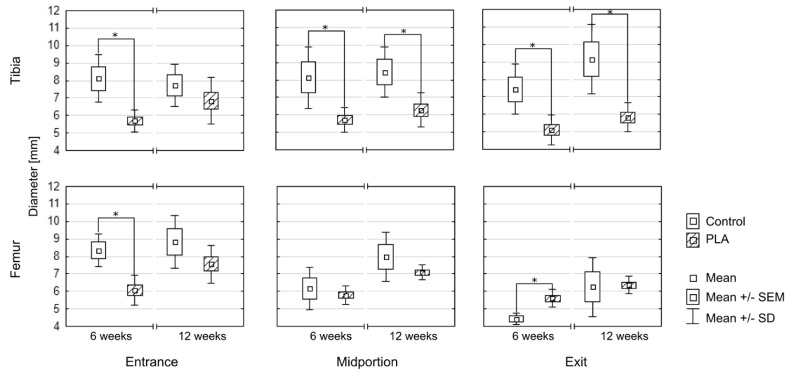
Comparison of bone tunnel diameters in the controls and PLA group; * statistically significant difference at *p* < 0.05.

Diameters of the bone tunnels after various healing time (6 and 12 weeks) are presented in Figure 8 and Table 1. At 6 weeks, the diameters of the tunnels in all segments except the midportion and exit of the femoral tunnel turned out to be significantly smaller in the PLA group than in the controls. At 12 weeks, the statistically significant between-group differences were observed solely for the midportion and exit of the tibial tunnel. While the diameters of most segments at 6 and 12 weeks did not differ significantly in the control group, a significant increase in all three diameters of the femoral tunnel was observed in the PLA group (Figure 9).

**Figure 10:**
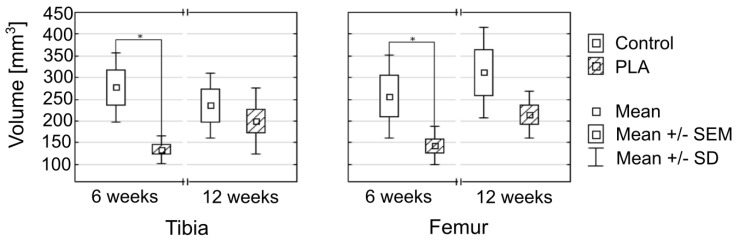
Comparison of bone tunnel volumes in the controls and PLA group at 6 and 12 weeks; * statistically significant difference at *p* < 0.05.

The results for the tunnel volume were consistent with those for the tunnel diameters (Figure 10, Table 1). In both tibia and femur, mean tunnel volumes at 6 weeks, but not at 12 weeks, were significantly smaller in the PLA group than in the controls. In line with those findings, a significant increase in the femoral tunnel volume and a tendency to increase in the tibial tunnel volume were observed at 12 weeks in the PLA group but not in the control group.

### 3.2. Bone Microstructure

**Figure 11:**
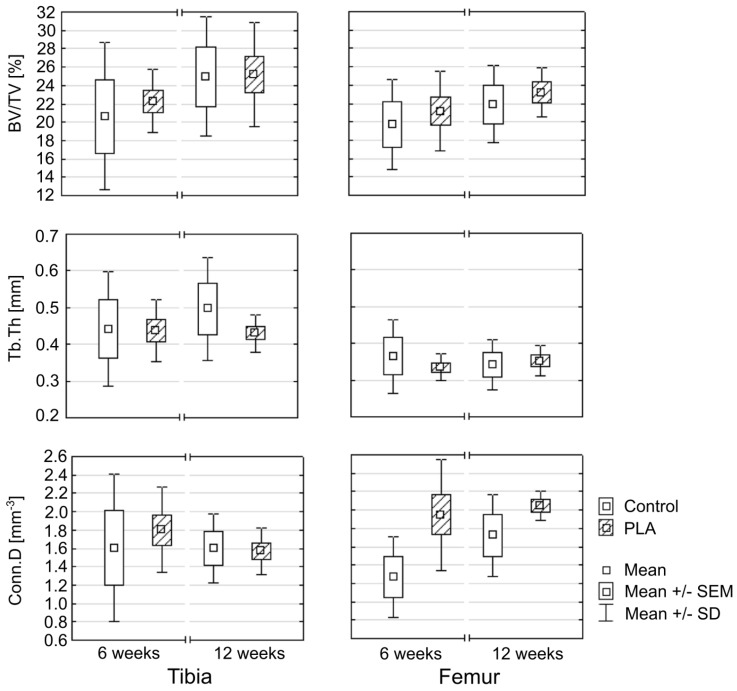
Comparison of histomorphometric parameters.

While no statistically significant differences were found in the microstructure of adjacent bone in the PLA and control group (*p* < 0.05), a tendency to better outcomes was observed in the former. Specifically, BV/TV values in the control group tended to be lower than in the PLA group (Figure 11, Table 2). In both groups, the values of the BV/TV tended to increase with the time elapsed since the reconstruction. However, considerable variance in BV/TV values was observed, especially in the controls, as shown by relatively high standard deviations.

While in the PLA group, Tb.Th values for the bone adjacent to the tibial and femoral tunnels were essentially the same at 6 and 12 weeks post-reconstruction, higher variance in the trabecular thickness at various study time points was observed in the control group (Table 2).

At 6 weeks after the reconstruction, Conn.D values for the tibial tunnel in the PLA group tended to be higher than in the controls, whereas virtually no difference in Conn.D values for the tibial tunnel in the PLA and control group was observed at 12 weeks. Regardless of the study timepoint, a trend towards a between-group difference in Conn.D values for the femoral tunnel was observed in favor of the PLA group (Table 2).

### 3.3. Three-Dimensional Visualization

The microstructure of bone remodeling is depicted in Figure 8. As shown in the figure, the edges of the bone tunnel in the PLA group were smooth, and no evidence of a non-uniform tunnel enlargement was observed. Also, the formation of the callus at both study timepoints seemed to be better in the PLA group than in the controls (Figure 8a,b). Regardless of the study group, the remodeling process was more advanced at 12 weeks post-reconstruction, which is consistent with the increase in BV/TV values.

## 4. Discussion

Early osteointegration of the tendon graft is crucial for successful rehabilitation and return to normal activities of daily living. The aim of this study was to improve the quality of tendon-bone fusion after ACL reconstruction. However, rather than focusing on soft tissues, such as the tendon, or mechanical properties of the tendon-stent-bone system, the study centered around interactions between the stent and the bone, especially bone remodeling after implantation of the stent. In the earlier animal study using a finite element analysis, we demonstrated that placement of the stent between the bone and the graft had a beneficial effect on the stress-strain response (See: Appendix A). The PLA tube was designated to act as a “shock absorber and stabilizer” during the ligament movements, preventing hypermobility of the graft within the bone tunnel. The numerical analysis demonstrated that the insertion of the stent contributed to a more favorable distribution of forces and lesser strain within the bone tunnel and created uniform conditions at the whole tunnel length. Based on those findings, we hypothesized that the implant not only acted as a scaffold for bone cells but might also protect the graft during the implantation and prevent the undesirable tunneling phenomenon. The aim of our present study was to verify this hypothesis in an animal model.

In our present study, we analyzed the influence of the PLA tube on bone tunnel remodeling using high-resolution micro-CT imaging; this enabled us to determine a set of objective parameters, such as tunnel diameter and volume, as well as histomorphometric characteristics of adjacent bone, such as BV/TV, Tb.Th., and Conn.D. The three-dimensionally molded stent was used as a buffer to minimize or eliminate undesired effects associated with post-traumatic or post-operative bone damage. Moreover, the stent was intended to act as a “sealant” filling the space between the tendon graft and bone tunnel wall, and hence, preventing penetration of synovial fluid into the tunnel. This would minimize the unfavorable effects of proinflammatory cytokines contained in the synovial fluid on bone remodeling, especially the BTE phenomenon.

We analyzed the diameters of the bone tunnels as a marker of potential BTE and a measure of the PLA tube effect on bone healing. Our findings suggested that the use of the PLA implant might not prevent the BTE completely, as the diameters of the femoral tunnel increased with the time elapsed since the reconstruction procedure. However, it needs to be stressed that no significant changes in the diameters of the tibial tunnel were observed over time in the PLA group. BTE might be a consequence of too early load of the operated limb, as the study animals did not undergo any controlled post-procedure rehabilitation, and the only factor limiting their activity was postoperative pain. However, it should be stressed that according to some rehabilitation protocols, human patients after ACL reconstruction are expected to weight-bear the operated extremity early after the procedure and to walk without crutches or braces. Nevertheless, our study showed that implantation of the PLA tube prevented the excessive widening of the bone tunnels.

Furthermore, a comparative analysis of femoral tunnel diameters in three segments (entry, midportion, and exit) demonstrated that in the control group, the tunnel tended to be wider at its entry. This might be a consequence of synovial fluid penetration to this segment of the tunnel and the detrimental effect of proinflammatory cytokines contained in the fluid on bone remodeling. In turn, the diameters of the three segments of the femoral tunnel in the PLA group were essentially the same, implying that insertion of the PLA tube might prevent the excessive inflow of the synovial fluid and minimize the harmful effects of proinflammatory cytokines.

To provide a better insight into the role of the PLA stent in bone remodeling after ACL reconstruction, we also analyzed the microstructure of adjacent bone. While the PLA group and the controls did not differ significantly in terms of their BV/TV values, the analysis of the latter in conjunction with Conn.D suggested that the use of the PLA stent might promote the faster formation of new bone. An increase in trabecular thickness and connectivity constitutes a response to mechanical overload [52]. After insertion of the PLA tube into the bone tunnel, some mechanical loads might be absorbed by the stent, rather than being directly delivered to the bone. Due to the resultant lesser mechanical load, the trabecular thickness in the PLA group did not increase significantly over time and was essentially the same at both 6 and 12 weeks post-reconstruction. This implied that the PLA stent prevented a non-uniform distribution of forces within the bone tunnel, creating a more stable environment for new bone formation. In contrast, variance in the trabecular thickness in the control group increased considerably with the time elapsed since the reconstruction procedure. The beneficial effect of the PLA stent on the remodeling of the adjacent bone was also confirmed by a tendency to higher connectivity density, especially around the femoral tunnel. Higher connectivity density reflected a larger number of connections between the trabeculae in the PLA group. Furthermore, the porosity of 45% and perforations of the stent could promote the accumulation of biological material from the bone marrow blood in the form of incubation over the entire surface of the stent and support bone tissue overgrowth.

### Strengths and Limitations

Our present study has several strengths and limitations. We proposed a new, accurate method to analyze bone remodeling after ACL reconstruction. Specifically, we resliced high-resolution micro-CT images to obtain accurate measurements in axial cross-sections. The results of previous studies, in which the images were not adjusted by reslicing [53,54], might have been biased due to a slight axial displacement of the examined cross-sections. In our opinion, this could have a considerable impact on the results, as the measurements of cross-sections that are not perfectly perpendicular might be slightly inaccurate. To improve the reproducibility of the measurements, we applied automatic thresholding (maximum entropy method); as a result, segmentation of the images was independent of the observer, making the results more reliable. One potential limitation of this study might be a too small number of examined sheep and a disproportion in the number of animals included in the PLA and control group (16 and 8, respectively). However, even in such a small sample, we found significant intergroup differences, confirming the beneficial effect of the PLA tube on bone healing. Another potential limitation of this study might be a relatively short follow-up. According to literature, full integration of the graft after ACL reconstruction takes longer than 12 weeks [55,56], and an increase in bone tunnel diameter and BTE phenomenon might be observed even up to 12 months post-reconstruction [57,58]. Nevertheless, the aim of our study was to analyze the changes that occurred early after the reconstruction rather than the long-term outcomes of the procedure. Finally, interpreting the hereby presented findings, one should remember that the sheep included in this study weight-bore their operated limbs as soon as they recovered from anesthesia, whereas human patients after ACL reconstruction are mobilized gradually and fully weight-bear their extremities even several weeks after the procedure.

## 5. Conclusions

The results of this study suggested that the application of a bioresorbable PLA tube might facilitate osteointegration of the tendon graft after the ACL reconstruction. The use of the bioresorbable PLA stent might partially prevent BTE and stimulate new bone formation, as shown by changes in BV/TV and Conn.D values. The beneficial effects of the PLA tube were likely associated with osteogenic and osteoconductive properties of polylactide. Our findings implied that the PLA tube might positively affect the quality of the graft-bone interface within the tibial and femoral tunnels, eventually resulting in better osteointegration of the ACL graft.

## Figures and Tables

**Table 1:** Morphometric parameters of tibial and femoral tunnels at 6 and 12 weeks post-ACL (anterior cruciate ligament).

Variable	Tibia			Femur		
	6 Weeks	12 Weeks	*p*-Value	6 Weeks	12 Weeks	*p*-Value
**Tunnel Entry Diameter**						
Controls	8.1 ± 1.3	7.7 ± 1.2	0.665006	8.3 ± 0.9	8.8 ± 1.5	0.665006
PLA group	5.7 ± 0.6	6.8 ± 1.3	0.156255	6.1 ± 0.9	7.6 ± 1.1	0.017673
*p*-value	0.008475	0.350238		0.008475	0.298618	
**Tunnel Midportion Diameter**						
Controls	8.1 ± 1.8	8.5 ± 1.5	0.885234	6.2 ± 1.2	8.0 ± 1.4	0.193932
PLA group	5.7 ± 0.7	6.3 ± 1.0	0.270149	5.8 ± 0.5	7.1 ± 0.4	0.003167
*p*-value	0.021857	0.021857		0.552215	0.219304	
**Tunnel Exit Diameter**						
Controls	7.4 ± 1.4	9.2 ± 2.0	0.193932	4.4 ± 0.3	6.3 ± 1.7	0.112352
PLA group	5.1 ± 0.8	5.8 ± 0.8	0.103563	5.6 ± 0.5	6.4 ± 0.5	0.032278
*p*-value	0.008475	0.033753		0.008475	0.924719	
**Tunnel Volume**						
Controls	277.6 ± 80.3	235.5 ± 75.0	0.470487	257.2 ± 95.5	311.5 ± 104.2	0.470487
PLA group	133.9 ± 31.1	200.4 ± 75.6	0.083124	142.6 ± 44.4	213.8 ± 53.9	0.033161
*p*-value	0.008475	0.444697		0.033753	0.240956	

**Table 2:** Microstructural parameters of tibial and femoral tunnels at 6 and 12 weeks post-ACL.

Variable	Tibia			Femur		
	6 Weeks	12 Weeks	*p*-Value	6 Weeks	12 Weeks	*p*-Value
BV/TV						
Controls	20.63 ± 8.04	24.98 ± 6.50	0.470487	19.73 ± 4.90	21.90 ± 4.22	0.470487
PLA group	22.30 ± 3.42	26.51 ± 4.81	0.103563	21.15 ± 4.36	23.22 ± 2.73	0.245279
*p*-value	0.671126	0.552215		0.932324	0.749119	
Tb.Th						
Controls	0.44 ± 0.16	0.50 ± 0.37	0.665006	0.37±0.10	0.34 ± 0.07	0.885234
PLA group	0.44 ± 0.09	0.45 ± 0.05	0.494837	0.34±0.04	0.35 ± 0.04	0.332922
*p*-value	0.798907	0.932324		0.671126	0.594033	
Conn.D						
Controls	1.61 ± 0.81	1.63 ± 0.14	0.885234	1.27 ± 0.44	1.72 ± 0.45	0.312322
PLA group	1.80 ± 0.46	1.57 ± 0.25	0.156255	1.95 ± 0.61	2.05 ± 0.16	0.948533
*p*-value	0.671126	0.932324		0.074532	0.240956	

**Figure A1:**
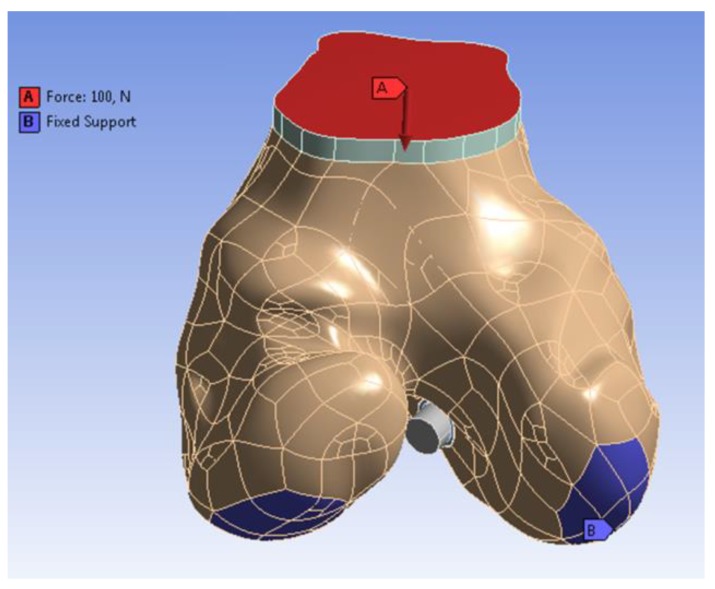
Application of force and fixed support to the femur model.

## Appendix A

### Appendix A.1. Construction of a Three-Dimensional Finite Element Model

The geometry of sheep femoral bone was modeled by the use of the advanced integrated CAx system (CATIA v5). During the numerical experiment, the levels of stress and strain were calculated for the two configurations: 1) cortical bone-cancellous bone stent-graft, and 2) cortical bone-cancellous bone graft.

To perform numerical analysis, a model of sheep posterior femur was created and used to simulate the postoperative load of the lower limb. The three-dimensional model, including both cortical bone (approximately 2.4 mm thick) and cancellous bone, was designed with CATIA v5 software. The model had a bone tunnel for graft implantation (with 4.5 mm in diameter, which corresponded to the maximum outer diameter of the stent used in the proper experiment). The model was cut, so the cortical bone was exposed, and the force corresponding to 1/4 of the sheep’s body weight was applied, with a resultant load of 100 N. During the experiment, the model was fixed on its medial and lateral condyle (Figure A1).

Material properties of the blood, cortical, and cancellous bone tissue used during the numerical study of fluid flow are summarized in Table A1. The calculations were carried out for the following mechanical properties of polylactide (LATI Latigea B01 F1 Bio-Polymer): density = 1.26 g/cm^3^, tensile strength = 55 MPa, modulus of elasticity = 3 GPa.

**Table A1:** Material properties of the blood, cortical, and cancellous bone.

Material Properties	Blood	Cortical Bone	Cancellous Bone
Density (kg/cm^3^)	1060	-	-
Proper heat (J/kg*K)	1006.43	-	-
Thermal conductivity (W/m*K)	0.0242	-	-
Viscosity (kg/m*s)	0.003	-	-
Input speed (mm/s)	1	-	-
Young’s modulus (MPA)	-	17,000	300
Density (g/cm^3^)	-	1.9	0.46
Poisson’s ratio	-	0.3	0.3
Compressive strength (MPa)	-	200	6

### Appendix A.2. Stress-Strain Analysis

The numerical simulation demonstrated that the stress was transmitted primarily through the cortical layer of the bone, which is consistent with the real-life conditions (Figure A2). The levels of stress in the graft and stent were small, no greater than several tens of Pa. Hence, to better visualize the stress levels, the scale was changed, so the red color corresponded to >100 Pa tension. The stress-strain analysis showed that implantation of the stent had a particular effect on stress distribution within the cortical bone and stent/graft interface (Figure A3). The analysis demonstrated that the distribution of the strain within the graft was more favorable after implantation of the stent (Figure A4 and Figure A5). This implied that implantation of the stent was not associated with additional stress for the graft. Theoretically, a decrease in the strain at the bone/graft interface observed after implantation of the stent should contribute to better ingrowth of the graft and reduce the likelihood of tunnel widening.

## References

[1] Kuskucu S.M. (2008). Comparison of short-term results of bone tunnel enlargement between EndoButton CL and cross-pin fixation systems after chronic anterior cruciate ligament reconstruction with autologous quadrupled hamstring tendons. J. Int. Med. Res..

[2] Moisala A.S., Järvelä T., Paakkala A., Paakkala T., Kannus P., Järvinen M. (2008). Comparison of the bioabsorbable and metal screw fixation after ACL reconstruction with a hamstring autograft in MRI and clinical outcome: A prospective randomized study. Knee Surg. Sports Traumatol. Arthrosc..

[3] Siebold R., Kiss Z.S., Morris H.G. (2008). Effect of compaction drilling during ACL reconstruction with hamstrings on postoperative tunnel widening. Arch. Orthop. Trauma Surg..

[4] Jaureguito J.W., Paulos L.E. (1996). Why grafts fail. Clin. Orthop. Relat. Res..

[5] Meyers A.B., Haims A.H., Menn K., Moukaddam H. (2010). Imaging of anterior cruciate ligament repair and its complications. AJR Am. J. Roentgenol..

[6] Beynnon B.D., Johnson R.J., Abate J.A., Fleming B.C., Nichols C.E. (2005). Treatment of anterior cruciate ligament injuries, part 2. Am. J. Sports Med..

[7] Beynnon B.D., Johnson R.J., Abate J.A., Fleming B.C., Nichols C.E. (2005). Treatment of anterior cruciate ligament injuries, part I. Am. J. Sports Med..

[8] Sundar S., Pendegrass C.J., Blunn G.W. (2009). Tendon bone healing can be enhanced by demineralized bone matrix: A functional and histological study. J. Biomed. Mater. Res. B Appl. Biomater..

[9] Chen C.H. (2009). Strategies to enhance tendon graft-bone healing in anterior cruciate ligament reconstruction. Chang Gung Med. J..

[10] Chen C.H., Chang C.H., Su C.I., Wang K.C., Liu H.T., Yu C.M., Wong C.B., Wang I.C. (2010). Arthroscopic single-bundle anterior cruciate ligament reconstruction with periosteum-enveloping hamstring tendon graft: Clinical outcome at 2 to 7 years. Arthroscopy.

[11] Chen C.H., Chen W.J., Shih C.H., Chou S.W. (2004). Arthroscopic anterior cruciate ligament reconstruction with periosteum-enveloping hamstring tendon graft. Knee Surg. Sports Traumatol. Arthrosc..

[12] Karaoglu S., Celik C., Korkusuz P. (2009). The effects of bone marrow or periosteum on tendon-to-bone tunnel healing in a rabbit model. Knee Surg. Sports Traumatol. Arthrosc..

[13] Rodeo S.A., Suzuki K., Deng X.H., Wozney J., Warren R.F. (1999). Use of recombinant human bone morphogenetic protein-2 to enhance tendon healing in a bone tunnel. Am. J. Sports Med..

[14] Baxter F.R., Bach J.S., Detrez F., Cantournet S., Corté L., Cherkaoui M., Ku D.N. (2010). Augmentation of bone tunnel healing in anterior cruciate ligament grafts: Application of calcium phosphates and other materials. J. Tissue Eng..

[15] Soon M.Y., Hassan A., Hui J.H., Goh J.C., Lee E.H. (2007). An analysis of soft tissue allograft anterior cruciate ligament reconstruction in a rabbit model: A short-term study of the use of mesenchymal stem cells to enhance tendon osteointegration. Am. J. Sports Med..

[16] Yeh W.L., Lin S.S., Yuan L.J., Lee K.F., Lee M.Y., Ueng S.W. (2007). Effects of hyperbaric oxygen treatment on tendon graft and tendon-bone integration in bone tunnel: Biochemical and histological analysis in rabbits. J. Orthop. Res..

[17] Yamazaki S., Yasuda K., Tomita F., Tohyama H., Minami A. (2005). The effect of transforming growth factor-beta1 on intraosseous healing of flexor tendon autograft replacement of anterior cruciate ligament in dogs. Arthroscopy.

[18] Sasaki K., Kuroda R., Ishida K., Kubo S., Matsumoto T., Mifune Y., Kinoshita K., Tei K., Akisue T., Tabata Y. (2008). Enhancement of tendon-bone osteointegration of anterior cruciate ligament graft using granulocyte colony-stimulating factor. Am. J. Sports Med..

[19] Darabos N., Haspl M., Moser C., Darabos A., Bartolek D., Groenemeyer D. (2011). Intraarticular application of autologous conditioned serum (ACS) reduces bone tunnel widening after ACL reconstructive surgery in a randomized controlled trial. Knee Surg. Sports Traumatol. Arthrosc..

[20] Gokce A., Beyzadeoglu T., Ozyer F., Bekler H., Erdogan F. (2009). Does bone impaction technique reduce tunnel enlargement in ACL reconstruction?. Int. Orthop..

[21] Höher J., Livesay G.A., Ma C.B., Withrow J.D., Fu F.H., Woo S.L. (1999). Hamstring graft motion in the femoral bone tunnel when using titanium button/polyester tape fixation. Knee Surg. Sports Traumatol. Arthrosc..

[22] Höher J., Scheffler S.U., Withrow J.D., Livesay G.A., Debski R.E., Fu F.H., Woo S.L. (2000). Mechanical behavior of two hamstring graft constructs for reconstruction of the anterior cruciate ligament. J. Orthop. Res..

[23] Iorio R., Vadalà A., Argento G., Di Sanzo V., Ferretti A. (2007). Bone tunnel enlargement after ACL reconstruction using autologous hamstring tendons: A CT study. Int. Orthop..

[24] Jagodzinski M., Foerstemann T., Mall G., Krettek C., Bosch U., Paessler H.H. (2005). Analysis of forces of ACL reconstructions at the tunnel entrance: Is tunnel enlargement a biomechanical problem?. J. Biomech..

[25] L’Insalata J.C., Klatt B., Fu F.H., Harner C.D. (1997). Tunnel expansion following anterior cruciate ligament reconstruction: A comparison of hamstring and patellar tendon autografts. Knee Surg. Sports Traumatol. Arthrosc..

[26] Agarwal S. (2004). Osteolysis—Basic science, incidence and diagnosis. Curr. Orthop..

[27] Irie K., Uchiyama E., Iwaso H. (2003). Intraarticular inflammatory cytokines in acute anterior cruciate ligament injured knee. Knee.

[28] Zysk S.P., Fraunberger P., Veihelmann A., Dörger M., Kalteis T., Maier M., Pellengahr C., Refior H.J. (2004). Tunnel enlargement and changes in synovial fluid cytokine profile following anterior cruciate ligament reconstruction with patellar tendon and hamstring tendon autografts. Knee Surg. Sports Traumatol. Arthrosc..

[29] Darabos N., Hundric-Haspl Z., Haspl M., Markotic A., Darabos A., Moser C. (2009). Correlation between synovial fluid and serum IL-1beta levels after ACL surgery-preliminary report. Int. Orthop..

[30] Clatworthy M.G., Annear P., Bulow J.U., Bartlett R.J. (1999). Tunnel widening in anterior cruciate ligament reconstruction: A prospective evaluation of hamstring and patella tendon grafts. Knee Surg. Sports Traumatol. Arthrosc..

[31] Iorio R., Vadalà A., Di Vavo I., De Carli A., Conteduca F., Argento G., Ferretti A. (2008). Tunnel enlargement after anterior cruciate ligament reconstruction in patients with post-operative septic arthritis. Knee Surg. Sports Traumatol. Arthrosc..

[32] Södergård A., Stolt M., Auras R., Lim L.T., Selke S.E.M., Tsuji H. (2010). Industrial Production of High Molecular Weight Poly (Lactic Acid). Poly (Lactic Acid). Synthesis, Structures, Properties, Processing, and Application.

[33] Li S.M., Garreau H., Vert M. (1990). Structure Property Relationships in the Case of the Degradation of Massive Aliphatic Poly-(Alpha-Hydroxy Acids) in Aqueous-Media. J. Mater. Sci. Mater. Med..

[34] Gorrasi G., Pantani R. (2013). Effect of PLA grades and morphologies on hydrolytic degradation at composting temperature: Assessment of structural modification and kinetic parameters. Polym. Degrad. Stab..

[35] Hakim R.H., Cailloux J., Santana O.O., Bou J., Sánchez-Soto M., Odent J., Raquez J.M., Dubois P., Carrasco F., Maspoch M.L. (2017). PLA/SiO_2_ composites: Influence of the filler modifications on the morphology, crystallization behavior, and mechanical properties. J. Appl. Polym. Sci..

[36] Gleadall A., Pan J., Kruft M.A., Kellomäki M. (2014). Degradation mechanisms of bioresorbable polyesters. Part 1.Effects of random scission, end scission and autocatalysis. Acta Biomater..

[37] Rapacz-Kmita K., Stodolak-Zych E., Szaraniec B., Gajek M., Dudek P. (2015). Effect of clay mineral on the accelerated hydrolytic degradation of polylactide in the polymer/clay nanocomposites. Mater. Lett..

[38] Ficek K., Stodolak E., Tomczak A., Stolarz M. (2012). Bioresorbable Polylactide Implant used in orthopaedic surgery—Case report. PrzypadkiMedyczne.pl.

[39] Gugala Z., Gogolewski S. (2005). The in vitro growth and activity of sheep osteoblasts on three-dimensional scaffolds from poly(L/DL-lactide) 80/20%. J. Biomed. Mater. Res. A.

[40] Gugala Z., Gogolewski S. (2004). Differentiation, growth and activity of rat bone marrow stromal cells on resorbable poly(L/DL-lactide) membranes. Biomaterials.

[41] Gugala Z., Lindsey R.W., Gogolewski S. (2007). New approaches in the treatment of critical-size segmental defects in long bones. Macromol. Symp..

[42] Leiggener C.S., Curtis R., Müller A.A., Pfluger D., Gogolewski S., Rahn B.A. (2006). Influence of copolymer composition of polylactide implants on cranial bone regeneration. Biomaterials.

[43] Ficek K., Wieczorek J., Stodolak-Zych E., Kosenyuk Y. (2012). A revised surgical concept of anterior cruciate ligament replacement in a rabbit model. Eng. Biomater..

[44] Nuss K.M., Auer J.A., Boos A., von Rechenberg B. (2006). An animal model in sheep for biocompatibility testing of biomaterials in cancellous bones. BMC Musculoskelet. Disord..

[45] Turner A.S. (2002). The sheep as a model for osteoporosis in humans. Vet. J..

[46] Stodolak-Zych E., Szumera M., Błażewicz M. (2013). Osteoconductive nanocomposite materials for bone regeneration. Mater. Sci. Forum.

[47] Stodolak-Zych E., Łuszcz A., Menaszek E., Ścisłowska-Czarencka A. (2014). Resorbable polymer membranes for medical applications. J. Biomim. Biomater. Tissue Eng..

[48] Schneider C.A., Rasband W.S., Eliceiri K.W. (2012). NIH Image to ImageJ: 25 years of image analysis. Nat. Methods.

[49] Skyscan N.V. (1987). Structural Parameters Measured by the Skyscan CT-Analyser Software.

[50] Doube M., Kłosowski M.M., Arganda-Carreras I., Cordelières F.P., Dougherty R.P., Jackson J.S., Schmid B., Hutchinson J.R., Shefelbine S.J. (2010). BoneJ: Free and extensible bone image analysis in ImageJ. Bone.

[51] Limaye A., Stock S.R. (2012). Drishti: A volume exploration and presentation tool. Developments in X-ray Tomography VIII, Proceedings of the SPIE Optical Engineering + Applications, San Diego, CA, USA, 12–16 August 2012.

[52] Ruimerman R., Hilbers P., van Rietbergen B., Huiskes R. (2005). A theoretical framework for strain-related trabecular bone maintenance and adaptation. J. Biomech..

[53] Iorio R., Di Sanzo V., Vadalà A., Conteduca J., Mazza D., Redler A., Bolle G., Conteduca F., Ferretti A. (2013). ACL reconstruction with hamstrings: How different technique and fixation devices influence bone tunnel enlargement. Eur. Rev. Med. Pharmacol. Sci..

[54] Silva A., Sampaio R., Pinto E. (2010). Femoral tunnel enlargement after anatomic ACL reconstruction: A biological problem?. Knee Surg. Sports Traumatol. Arthrosc..

[55] Weiler A., Hoffmann R.F., Bail H.J., Rehm O., Südkamp N.P. (2002). Tendon healing in a bone tunnel. Part II: Histologic analysis after biodegradable interference fit fixation in a model of anterior cruciate ligament reconstruction in sheep. Arthroscopy.

[56] Weiler A., Peine R., Pashmineh-Azar A., Abel C., Südkamp N.P., Hoffmann R.F. (2002). Tendon healing in a bone tunnel. Part I: Biomechanical results after biodegradable interference fit fixation in a model of anterior cruciate ligament reconstruction in sheep. Arthroscopy.

[57] Buelow J.U., Siebold R., Ellermann A. (2002). A prospective evaluation of tunnel enlargement in anterior cruciate ligament reconstruction with hamstrings: Extracortical versus anatomical fixation. Knee Surg. Sports Traumatol. Arthrosc..

[58] Vadalà A., Iorio R., De Carli A., Argento G., Di Sanzo V., Conteduca F., Ferretti A. (2007). The effect of accelerated, brace free, rehabilitation on bone tunnel enlargement after ACL reconstruction using hamstring tendons: A CT study. Knee Surg. Sports Traumatol. Arthrosc..

